# Genome-Wide Chromatin Immunoprecipitation Sequencing Analysis of the *Penicillium chrysogenum* Velvet Protein PcVelA Identifies Methyltransferase PcLlmA as a Novel Downstream Regulator of Fungal Development

**DOI:** 10.1128/mSphere.00149-16

**Published:** 2016-07-13

**Authors:** Kordula Becker, Sandra Ziemons, Katharina Lentz, Michael Freitag, Ulrich Kück

**Affiliations:** a Lehrstuhl für Allgemeine und Molekulare Botanik, Ruhr-Universität Bochum, Bochum, Germany; b Department of Biochemistry and Biophysics, Oregon State University, Corvallis, Oregon, USA; Karlsruhe Institute of Technology (KIT), Institute for Applied Biosciences

**Keywords:** ChIP-seq, PcLlmA, PcVelA, *Penicillium chrysogenum*, methyltransferase, protein-DNA interactions, velvet complex

## Abstract

*Penicillium chrysogenum* is the sole industrial producer of the β-lactam antibiotic penicillin, which is the most commonly used drug for treating bacterial infections. In *P. chrysogenum* and other filamentous fungi, secondary metabolism and morphogenesis are controlled by the highly conserved multisubunit velvet complex. Here we present the first chromatin immunoprecipitation next-generation sequencing (ChIP-seq) analysis of a fungal velvet protein, providing experimental evidence that a velvet homologue in *P. chrysogenum* (PcVelA) acts as a direct transcriptional regulator at the DNA level in addition to functioning as a regulator at the protein level in *P. chrysogenum*, which was previously described. We identified many target genes that are related to processes known to be dependent on PcVelA, e.g., secondary metabolism as well as asexual and sexual development. We also identified seven PcVelA target genes that encode putative methyltransferases. Yeast two-hybrid and bimolecular fluorescence complementation analyses showed that one of the putative methyltransferases, PcLlmA, directly interacts with PcVelA. Furthermore, functional characterization of PcLlmA demonstrated that this protein is involved in the regulation of conidiosporogenesis, pellet formation, and hyphal morphology, all traits with major biotechnological relevance.

**IMPORTANCE** Filamentous fungi are of major interest for biotechnological and pharmaceutical applications. This is due mainly to their ability to produce a wide variety of secondary metabolites, many of which are relevant as antibiotics. One of the most prominent examples is penicillin, a β-lactam antibiotic that is produced on the industrial scale by fermentation of *P. chrysogenum*. In recent years, the multisubunit protein complex velvet has been identified as one of the key regulators of fungal secondary metabolism and development. However, until recently, only a little has been known about how velvet mediates regulation at the molecular level. To address this issue, we performed ChIP-seq (chromatin immunoprecipitation in combination with next-generation sequencing) on and follow-up analysis of PcVelA, the core component of the velvet complex in *P. chrysogenum*. We demonstrate direct involvement of velvet in transcriptional control and present the putative methyltransferase PcLlmA as a new downstream factor and interaction partner of PcVelA.

## Abstract (precis)

Filamentous fungi are of major interest for biotechnological and pharmaceutical applications. This is due mainly to their ability to produce a wide variety of secondary metabolites, many of which are relevant as antibiotics. One of the most prominent examples is penicillin, a β-lactam antibiotic that is produced on the industrial scale by fermentation of *P. chrysogenum*. In recent years, the multisubunit protein complex velvet has been identified as one of the key regulators of fungal secondary metabolism and development. However, until recently, only a little has been known about how velvet mediates regulation at the molecular level. To address this issue, we performed ChIP-seq (chromatin immunoprecipitation in combination with next-generation sequencing) on and follow-up analysis of PcVelA, the core component of the velvet complex in *P. chrysogenum*. We demonstrate direct involvement of velvet in transcriptional control and present the putative methyltransferase PcLlmA as a new downstream factor and interaction partner of PcVelA.

## INTRODUCTION

The discovery of β-lactam antibiotics has rightly been described as one of the most significant milestones in human history, as it enabled effective treatment of bacterial infections for the first time (1). Penicillin, the most commonly used drug within this group of antibiotics, is synthesized by the filamentous ascomycete *Penicillium chrysogenum*, which was first described in 1928 (2). Since then, immense efforts have been made to maximize penicillin yields in large-scale industrial production. For many years, strain improvement programs were based on random mutagenesis approaches, such as treatment with X rays, UV irradiation, and nitrogen mustard mutagenesis (1, 3, 4). One of the main drawbacks of random mutagenesis is that it introduces both desirable and undesirable mutations, and large-scale screening processes are needed to identify the mutant strains with the improved characteristics. Thus, one of the main goals of current strain improvement programs is to replace random mutagenesis with targeted genetic engineering approaches in order to speed and simplify the generation of new strains with properties that help optimize penicillin production. Hence, it is critical to understand the regulation of *P. chrysogenum* morphology, development, and secondary metabolism at the molecular level.

Secondary metabolism and differentiation processes in various filamentous fungi are orchestrated by the multisubunit velvet complex (5 – 7). The founding member of the complex, VeA (velvet A), was first described as a light-dependent regulator in *Aspergillus nidulans* (8). Subsequent characterization of *veA* deletion and overexpression mutants has confirmed its roles in the regulation of sexual and asexual development, morphogenesis, virulence, and secondary metabolism in several species (6, 7, 9 – 12). Deletion of the gene for the VeA homologue in *P. chrysogenum* (Pc*velA*) reduces penicillin production and causes light-independent formation of conidiospores. Moreover, the deletion results in dichotomous branching of hyphae and increases pellet formation in shaking cultures (6). Besides PcVelA, members of the velvet protein family in *P. chrysogenum* include PcVelB, PcVelC, and PcVosA (13). Furthermore, the putative *S*-adenosyl-l-methionine (SAM)-dependent methyltransferase (MTase) LaeA (loss of *aflR* expression A), which acts as a global regulator of secondary metabolism and development in various euascomycetes (6, 7, 14, 15), is also part of the velvet complex. According to our current working model, all of the velvet subunits, together with PcLaeA, can interact with at least one other velvet subunit (6, 13). Analyses of a comprehensive set of single- and double-deletion mutants have shown that PcVelA, together with PcLaeA and PcVelC, is an activator of penicillin biosynthesis, whereas PcVelB represses this process. Moreover, PcVelB and PcVosA promote conidiation, while PcVelC has an inhibitory effect (6, 13).

Almost 10 years ago, Ni and Yu postulated that velvet proteins might act as global transcriptional regulators, representing a new fungus-specific class of transcription factors (TFs) (16). Supporting evidence for this idea was provided by microarray analyses with *P. chrysogenum* that showed that PcVelA influences the expression of 13.6% of all nuclear genes (6). Further, recent RNA-sequencing analyses of *Aspergillus fumigatus* and *A. nidulans* revealed that, respectively, 32% and 26% of all protein-coding genes are regulated in a VeA-dependent manner (17). The first experimental evidence that velvet proteins act as direct regulators at the DNA level was provided for *Histoplasma capsulatum* (18). By chromatin immunoprecipitation with microarray technology (ChIP-chip), two distinct *cis*-acting regulatory sequences were identified, and these are bound directly by Ryp2 and Ryp3, two orthologs of VosA and VeA/VelB, respectively (18). This finding was further supported by structural analyses (19), demonstrating that the velvet domain acts as a DNA-binding domain in *A. nidulans*.

Although extensive efforts were made to decipher the molecular mechanisms that control velvet protein-mediated regulation in various fungi, these mechanisms remain poorly understood. Therefore, the aim of this work was to shed light onto PcVelA regulatory functions on a genome-wide scale by using ChIP combined with next-generation sequencing (ChIP-seq). Follow-up analyses were designed to further elucidate PcVelA DNA-binding properties on a molecular level and to enable functional characterization of new PcVelA downstream factors.

## RESULTS

### Generation of a genome-wide PcVelA DNA-binding profile.

Prior to carrying out ChIP experiments, we performed Northern blot hybridization to analyze expression of Pc*velA* under the control of its native promoter sequence and under conditions scheduled for ChIP. As only minimal amounts of the Pc*velA* transcript were detected in the wild-type P2niaD18 and ΔPc*ku70*-FRT2 strains (see Fig. S1A in the supplemental material), we decided to use a mutant strain carrying a P_gpd_::Pc*velA*::*egfp* fusion construct for further experiments (Fig. S1B). The corresponding plasmid, pPcVelA-EGFP, was ectopically integrated into *P. chrysogenum* ΔPc*velA*, a marker-free Pc*velA* deletion strain. Successful transformation and expression of Pc*velA*::*egfp* in strain PcVelA-ChIP was verified by PCR, Northern blot, and Western blot analyses (Fig. S1A, S1C, and S1D). Fluorescence microscopy was used to confirm both the presence and the nuclear localization of PcVelA-EGFP (Fig. S1E). The ChIP-seq experiments were performed using two independent biological samples obtained from shaking cultures of strain PcVelA-ChIP. The corresponding data sets were designated PcVelA_shaking_1 and PcVelA_shaking_2. For the input control sample, PcVelA_shaking_input, DNA from strain PcVelA-ChIP was isolated after cell lysis and fragmentation of chromatin by sonication but prior to chromatin immunoprecipitation. As the input DNA went through the whole experimental procedure without any specific enrichment, it represents fractionated genomic DNA. During the bioinformatics analysis, only regions that met the following criteria were regarded as specific PcVelA binding regions: (i) a ≥4-fold enrichment in ChIP DNA over input DNA (ii), a false discovery rate (FDR) threshold of ≤0.001, and (iii) a Poisson *P* value of ≤1.00e−04. We identified 764 and 1,001 regions that were specifically bound by PcVelA in the PcVelA_shaking_1 and PcVelA_shaking_2 data sets, respectively (Table 1).

**TABLE 1:** ChIP-seq design and results

Sample	No. of reads^a^	No. mapped^b^	% mapped^c^	No. of peaks whose FDR was ≤0.001^d^	No. of differential peaks^e^	No. of total peaks^f^	Estimated fragment length^g^
PcVelA_shaking_1	34,074,601	20,835,894	61.15	6,088	1,937	764	235
PcVelA_shaking_2	29,736,045	17,177,895	57.77	6,090	1,362	1,001	231
PcVelA_shaking_input	20,383,512	18,540,910	90.96				

a Total number of sequenced reads.

b Total number of reads mapped to the *P. chrysogenum* P2niaD18 genome.

c Fraction of tags found in peaks versus in the genomic background determined by HOMER.

d Number of peaks passing the FDR threshold of ≤0.001.

e Number of peak regions showing at least a 4-fold enrichment in the ChIP sample over the input DNA.

f Total number of peak regions after local background filtering and clonal filtering.

g Estimated fragment length used for sequencing determined from tag autocorrelation analysis.

10.1128/mSphere.00149-16.1 Figure S1  Construction of PcVelA-ChIP strains. (A) Northern blot analysis revealed low expression of Pc*velA* under the control of its native promoter sequence and under conditions scheduled for ChIP in wild-type P2niaD18 and ΔPc*ku70* FRT2 strains. The ΔPc*velA* and PcVelA-ChIP strains are shown as controls. (B) Plasmid pPcVelA-EGFP, harboring a P_gpd_::Pc*velA*::*egfp* fusion construct, was used for ectopic integration into the ΔPc*velA* strain, a marker-free Pc*velA* deletion strain. (C) PCR analysis confirmed integration of P_gpd_::Pc*velA*::*egfp*. Binding positions of primers PcvelA_f and egfp_r are indicated as arrows in panel B. (D) Presence of the epitope-tagged protein PcVelA-EGFP in crude protein extract from strain PcVelA-ChIP was confirmed using SDS-PAGE and Western blot analysis. (E) Fluorescence microscopy confirmed the nuclear localization of PcVelA-EGFP in the PcVelA-ChIP strain. Strains were grown on solid medium for 48 h. Scale bar = 10 µm. Download Figure S1, TIF file, 8.9 MB. Copyright © 2016 Becker et al. 2016 Becker et al. This content is distributed under the terms of the Creative Commons Attribution 4.0 International license.

When we compared the two data sets, only peaks that had a maximum distance of 100 nucleotides (nt) were regarded as overlapping. Doing so, we identified 592 sites that were specifically bound by PcVelA in both biological replicates (see Data Set S1 in the supplemental material). As part of our initial analysis, the peak regions were classified according to their genomic location, i.e., as being close to or within open reading frames (ORFs). A total of 78.9% (467/592) of the peaks showed intergenic localization, while 21.1% (125/592) were located within protein-coding regions. Of the 467 peaks that showed intergenic localization, 39 were within the 3′ region of both adjacent ORFs, 225 were 5′ of one neighboring gene, and 203 were positioned within divergent promoter regions. Thus, considering only those genes located in the 5′-to-3′ orientation with regard to neighboring peak regions, we identified a total of 631 genes that might be controlled directly by PcVelA with high confidence and stringency. Putative binding sites in the 3′ region or coding regions may also be used (20), but these are not considered further here. Previous microarray analyses (6) confirmed that 18.9% (119/631) of these genes showed ≥2-fold PcVelA-dependent changes in expression. This overlap is consistent with previous ChIP-seq data from analyses of *P. chrysogenum*, *Saccharomyces* *cerevisiae*, and higher eukaryotes, which revealed overlaps of 10 to 50% between TF occupancy and the expression of neighboring genes (20–25).

10.1128/mSphere.00149-16.7 Data Set S1  Complete data set from bioinformatics analysis of PcVelA ChIP-seq data. Download Data Set S1, XLSX file, 0.3 MB. Copyright © 2016 Becker et al. 2016 Becker et al. This content is distributed under the terms of the Creative Commons Attribution 4.0 International license.

### Validation of the PcVelA ChIP-seq data.

In order to validate the biological significance of our data set and to rule out bias from the bioinformatics analysis, the PcVelA-specific enrichment of four selected target regions that were identified in the ChIP-seq analyses was confirmed using quantitative ChIP-PCR. Target regions were selected to cover a range of PcVelA binding sites, from high-affinity to midaffinity sites, as deduced from the ChIP-seq data. The enrichment of the target region was calculated as the ratio of the region of interest to the level of a control region showing no PcVelA-specific enrichment in ChIP DNA relative to this ratio in the input DNA. A randomly selected region that showed no specific enrichment in the ChIP-seq data was used as a control (NC). Data from the ChIP-PCR analysis were compared to peak values obtained in the bioinformatics analysis. These values represent the average number of sequence tags found within a peak region after normalization to a total of 10 million mapped tags. The ChIP-PCR results were consistent with the peak values, confirming the specific enrichment of all tested PcVelA target regions (Fig. S2).

10.1128/mSphere.00149-16.2 Figure S2  Validation of PcVelA ChIP-seq data. ChIP-PCR analysis was performed to analyze the enrichment of selected PcVelA target regions in ChIP DNA compared to input DNA. Enrichment was calculated as the ratio of the region of interest to a control region showing no PcVelA-specific enrichment in ChIP-seq experiments. Another region showing no PcVelA-specific enrichment in ChIP-seq analysis is shown as a control (NC). ChIP-PCR ratios (gray bars) are shown in comparison to the corresponding peak values, as obtained from bioinformatics analysis (black bars). Values for ChIP-PCR are the mean scores from three biological replicates; averages ± standard deviations are indicated. Peak regions are named according to neighboring genes (see Data Set S1). Download Figure S2, TIF file, 0.6 MB. Copyright © 2016 Becker et al. 2016 Becker et al. This content is distributed under the terms of the Creative Commons Attribution 4.0 International license.

### Categorization of putative PcVelA target genes.

Within our ChIP-seq data set, we identified a remarkably high number of putative PcVelA target genes that were directly related to cellular and developmental processes that are known to be controlled by velvet (Table 2). Most of these genes had shown PcVelA-dependent expression profiles in previous microarray analyses comparing expression levels in a Pc*velA* deletion strain to those in the corresponding ΔPc*ku70* wild-type strain (6). However, this was not the case for genes that control conidiation, since this developmental step is most probably regulated at later time points (~120 h). Prominent examples of direct PcVelA target genes include *con-6* (*Pc16g03240*), *flbC* (*Pc12g12190*), *flbD* (*Pc13g03170*), *artA* (*Pc18g03940*), and *brlA* (*Pc23g00400*), all of which are related to different developmental steps of conidiation (26 – 30). The putative target genes with functions that were related to spore viability and protection included *treA* or *ath1* (*Pc16g11870*), which encodes an α,α-trehalose glucohydrolase, *Pc16g06690*, which encodes a precursor of the spore wall fungal hydrophobin DewA, and *Pc13g09910*, which encodes a late-embryogenesis-abundant (LEA) domain protein (31 – 33). Interestingly, a PcVelA DNA-binding region was identified within the upstream region of Pc*velB*, a gene that encodes a component of the velvet complex that activates conidiospore formation in various filamentous fungi (5, 7, 13). However, Pc*velB* did not show PcVelA-dependent expression in previous microarray analyses, and the peak value of the corresponding ChIP-seq peak region suggested a rather low-affinity target region. In addition to identifying genes related to conidiation and development, we identified several genes that encoded proteins related to various aspects of secondary metabolism, such as *Pc21g08920*, which encodes a norsolorinic acid reductase, *Pc21g12630*, which encodes a nonribosomal peptide synthetase, and *stuA* (*Pc13g04920*), which encodes a TF with a basic helix-loop-helix domain. Notably, StuA not only regulates penicillin biosynthesis in *P. chrysogenum* (34) but also controls asexual reproduction, especially conidiophore development, in *A. nidulans* (35).

**TABLE 2:**
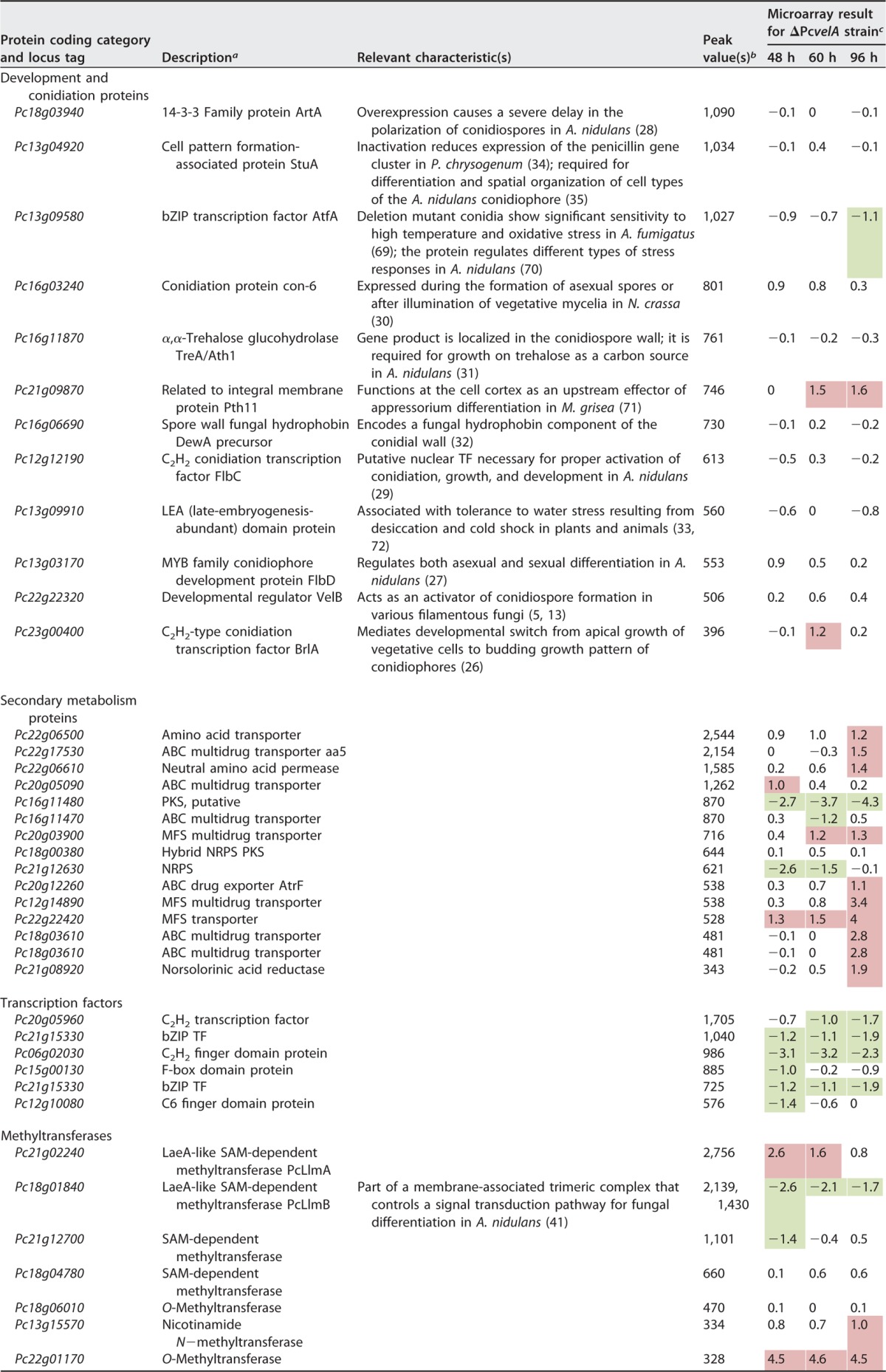
Selected PcVelA target genes identified in ChIP-seq analyses^d^

a As obtained from blastp analysis (http://blast.ncbi.nlm.nih.gov/Blast.cgi).

b The statistical peak value is the average tag count found at peak normalized to 10 million total mapped tags.

c Microarray data showing expressional changes in the ΔPc*velA* strain relative to expression in the wild-type ΔPc*ku70* strain after 48, 60, and 96 h of cultivation (6).

d For comparison, previously obtained expression data from microarray hybridizations (6) are shown. Upregulated genes are marked by red shading, and downregulated genes are marked by green shading. PcVelA target genes within each category are arranged based on peak values obtained from bioinformatics ChIP-seq analysis. High peak values indicate strong binding of PcVelA to the respective 5′ region. NRPS, nonribosomal peptide synthetase; PKS, polyketide synthase; MFS, major facilitator superfamily.

Genes that are related to known velvet-regulated functions were localized next to some of the peaks with the highest peak values. It is generally accepted that the regions that are identified by ChIP-seq analysis with DNA-binding proteins are positioned next to known functional target genes (36). However, ChIP-seq analysis also identified a large number of highly significant PcVelA target regions next to genes that have not previously been associated with PcVelA or with any other component of the velvet complex. We identified, among other genes, numerous genes that encode uncharacterized TFs, which may act as downstream factors of PcVelA. In Table 2, we included only those TF genes that showed PcVelA-dependent expression in a microarray analysis. This observation is consistent with ChIP-seq data obtained with the *Neurospora crassa* circadian regulator white collar complex (WCC), revealing that the protein directly controls the expression of 24 TFs, which in turn might be involved in transcriptional control on a second hierarchical level (20). Furthermore, we also identified seven genes encoding putative MTases.

### *De novo* prediction and validation of a PcVelA DNA-binding motif.

**FIG 1:**
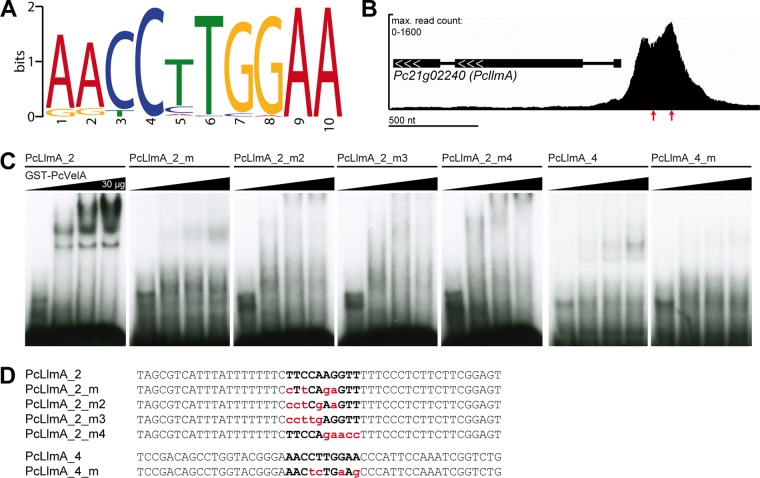
Electrophoretic mobility shift assays (EMSAs) confirm PcVelA binding to the predicted DNA-binding consensus sequence PcVelA.M1. (A) PcVelA-specific peak regions were submitted to MEME (63) for *de novo* motif prediction. Only the most significant putative DNA-binding motif, PcVelA.M1, is shown. The size of each letter is proportional to the frequency of each nucleotide at this position within the consensus sequence. (B) Enlargement of ChIP-seq profile from PcVelA_shaking_1 next to *Pc21g02240*, encoding the putative SAM-dependent MTase PcLlmA. Positions of oligonucleotides, which were used for shift analysis, are indicated by red arrows. (C) EMSAs were performed using 50-nt radiolabeled double-stranded oligonucleotide probes (PcLlmA_2, PcLlmA_4) derived from the Pc*llmA* promoter region and increasing amounts of purified GST-PcVelA_1–256_ protein. Application of mutated versions of PcLlmA_2 and PcLlmA_4 resulted in reduced formation of protein-DNA complexes. (D) Sequences of oligonucleotide probes used for the experiment whose results are shown in panel C. The PcVelA.M1 binding sequence is indicated with bold letters. Mutated bases are lowercase and red.

We used the MEME (Multiple Expression Motifs [EM] for Motif Elicitation) platform to perform *de novo* prediction of a PcVelA DNA-binding motif based on the peak regions obtained in the ChIP-seq experiments. Our analysis revealed one highly significant motif sequence, designated PcVelA.M1 (Fig. 1A), that was present in 275 (46.5%) out of 592 peak regions using a statistical threshold of a *P* of ≤0.001. While a comparison of PcVelA.M1 to known binding motifs in the JASPAR CORE (2014) fungi database did not reveal any significant matches, comparison to the JASPAR CORE (2014) vertebrate database revealed some interesting similarities to other motifs. As shown in Fig. S3 in the supplemental material, PcVelA.M1 most closely resembled the DNA-binding motifs of NR2E3, a TF involved in human photoreceptor development (37, 38), and NR2F1, a nuclear hormone receptor and transcriptional regulator that plays an important role in the neurodevelopment of the visual system in humans (39). Independently of the bioinformatics analysis, we also noticed strong similarity between PcVelA.M1 and the DNA-binding motif sequence described for the Ryp2/Ryp3 heterodimer from *H. capsulatum* (18) as well as weak similarity to a DNA-binding consensus sequence that was recently described for *A. nidulans* VosA (19).

10.1128/mSphere.00149-16.3 Figure S3  Comparison of PcVelA.M1 to the JASPAR CORE databases. The PcVelA.M1 motif sequence was submitted to TOMTOM (64), using default parameters. The top two matches to known DNA-binding motifs from vertebrates are given. The associated proteins, identifiers from the JASPAR CORE database, *P* values, and *E* values are indicated. The size of each letter is proportional to the frequency of each nucleotide at this position within the consensus sequence. Motifs are centered on common central nucleotides. Download Figure S3, TIF file, 6.6 MB. Copyright © 2016 Becker et al. 2016 Becker et al. This content is distributed under the terms of the Creative Commons Attribution 4.0 International license.

To further verify the biological significance of PcVelA.M1, we performed electrophoretic mobility shift assays (EMSAs) using a glutathione *S*-transferase (GST)-tagged version of the PcVelA N-terminal region from amino acids 1 to 256 (PcVelA_1–256_), which was purified from *Escherichia coli*, and 50-nt DNA sequences (PcLlmA_2 and PcLlmA_4) derived from a region within the Pc*llmA* upstream sequence (Fig. 1B). Both oligonucleotides contained exactly one copy of PcVelA.M1 and showed specific binding to PcVelA_1–256_ (Fig. 1C). Specific binding was also documented for full-length PcVelA (Fig. S4); however, the PcVelA N terminus seemed to be sufficient for effective DNA binding. As PcVelA_1–256_ includes the complete velvet domain, this might indicate that the velvet domain mediates DNA binding, as demonstrated for *A. nidulans* VosA and VelB (19). The specificity of the binding between PcVelA and the DNA-binding consensus sequence PcVelA.M1 was further verified by testing the binding of mutated versions of the PcLlmA_2 and PcLlmA_4 oligonucleotides (Fig. 1C and D) to PcVelA_1–256_. Formation of protein-DNA complexes was drastically reduced when mutations were introduced into the motif sequence. This observation supports our hypothesis that PcVelA.M1 is necessary and sufficient to mediate DNA binding of PcVelA to its specific target sites.

10.1128/mSphere.00149-16.4 Figure S4  EMSA using full-length PcVelA. EMSAs were performed using 50-nt radiolabeled double-stranded oligonucleotide probes (PcLlmA_2, PcLlmA_4) derived from the Pc*llmA* promoter region and increasing amounts of purified GST-PcVelA protein. Positions of free probe (*) and protein-DNA complexes (→) are indicated. Download Figure S4, TIF file, 0.7 MB. Copyright © 2016 Becker et al. 2016 Becker et al. This content is distributed under the terms of the Creative Commons Attribution 4.0 International license.

### Further characterization of putative MTases.

**FIG 2:**
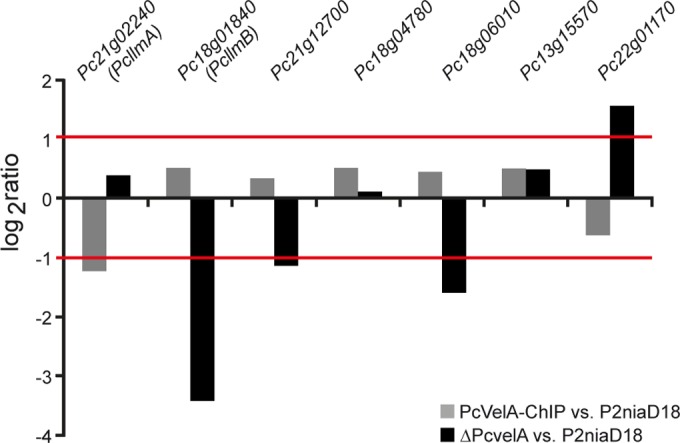
qRT-PCR analysis confirms PcVelA-dependent expression of putative MTase-encoding genes. Analysis of relative log_2_-fold gene expression ratios of putative MTase-encoding genes confirmed PcVelA dependency. Ratios of their expression in PcVelA-ChIP (gray bars) and the ΔPc*velA* strain (black bars) are shown compared to that in wild-type P2niaD18. Values are the mean scores from three biological replicates. Partial amplification of the 18S rRNA was used as a reference for normalization.

We found that five of the seven genes that encoded putative MTases in the PcVelA ChIP-seq data set showed significant PcVelA-dependent expression profiles in previous microarray analyses (Table 2) (6). Because recent reports from other euascomycetes point to a functional link between VeA homologs and several putative MTases (40 – 42), we focused further analyses on this group of new PcVelA target genes. First, we performed quantitative reverse transcription-PCR (qRT-PCR) analysis to validate the PcVelA-dependent expression of MTase-encoding genes. We compared expression levels in strain PcVelA-ChIP, which is characterized by elevated Pc*velA* expression, and the ΔPc*velA* strain with levels in wild-type P2niaD18. As a reference for normalization, we used amplification of a fragment of the 18S rRNA. All strains were grown under the same conditions as for ChIP-seq sample preparation. As shown in Fig. 2, PcVelA-dependent expression profiles were confirmed for four out of seven tested MTase genes, namely, Pc*llmA* (*Pc21g02240*), Pc*llmB* (*Pc18g01840*), *Pc21g12700*, and *Pc22g01170. Pc18g06010* showed PcVelA-dependent expression in qRT-PCR but not in microarray analysis (Table 2). Next, the amino acid sequences of the putative MTases were compared to the sequences of PcLaeA, a putative methyltransferase that is part of the velvet complex, as well as *A. nidulans* LlmF, VapB, and VipC (homolog of PcLlmB), which have previously been described as velvet-associated proteins (40, 41). In general, a set of three conserved sequence motifs (motifs I to III) that are essential for catalytic activity are found in most MTases (43, 44). The most prominent one, motif I, is characterized by the glycine (G)-rich sequence E/DXGXGXG, which is conserved in fungi, plants, and humans (41, 45, 46). Overall comparison of amino acid sequences revealed that PcLlmA, PcLlmB, and PcLaeA, as well as LlmF, VapB, and VipC from *A. nidulans*, had only moderate similarity (Fig. S5A). However, the region spanning SAM-binding motif I was highly conserved, as were several amino acids residues that are involved in SAM binding (45, 46). This degree of conservation is also found for other SAM-dependent MTases, which generally share a set of conserved MTase sequence motifs plus a highly conserved structural fold but show rather low overall sequence similarity (43, 44, 46).

10.1128/mSphere.00149-16.5 Figure S5  Multiple sequence alignments of putative SAM-dependent MTases and the secondary structure of PcLlmA. (A) Alignment of amino acid sequences revealed a high degree of conservation between PcLlmA, PcLlmB, and PcLaeA as well as *A. nidulans* LlmF, VipC, and VapB. Alignments were visualized using Jalview according to the ClustalX color scheme (http://www.jalview.org/). The secondary-structure model of PcLlmA was generated using the I-TASSER server (46–48) and is indicated under the primary sequence. The predicted SAM-binding motif D/EXGXGXG and conserved residues known to be involved in SAM binding (44, 45) are marked by dashed boxes. (B) The secondary structure of the putative SAM-dependent MTase PcLlmA was predicted using the I-TASSER (46–48) server. The protein shows a typical SAM-MTase fold, characterized by alternating β-strands (β1 to β7) and α-helices (α1 to α8), forming a seven-stranded β-sheet. α-Helices are shown in red, β-strands are shown in blue, and the highly conserved glycine-rich sequence E/DXGXGXG is marked in orange. The N and C termini of the protein are labeled. Download Figure S5, TIF file, 11.7 MB. Copyright © 2016 Becker et al. 2016 Becker et al. This content is distributed under the terms of the Creative Commons Attribution 4.0 International license.

### PcLlmA shows the typical SAM-MTase fold.

Because we identified Pc*llmA* as a direct target gene of PcVelA and because the SAM-binding domains of PcLlmA and other velvet-associated MTases were highly conserved (Fig. S5A), we continued to functionally characterize the protein. First the PcLlmA amino acid sequence was submitted to the I-TASSER server (47 – 49) to predict the secondary structure of the protein. As shown in Fig. S5B, PcLlmA has the typical SAM-dependent MTase fold, which is characterized by alternating α-helices (α1 to α8) and β-strands (β1 to β7) that form a seven-stranded β-sheet (46). The highly conserved glycine-rich sequence E/DXGXGXG (motif I) and the conserved amino acid residues that are involved in SAM binding (45, 46) localize to the SAM-binding N-terminal region of the protein, whereas the substrate-binding region of the protein is located at the C terminus of the β-sheet.

### PcLlmA directly interacts with PcVelA in the nucleus.

**FIG 3:**
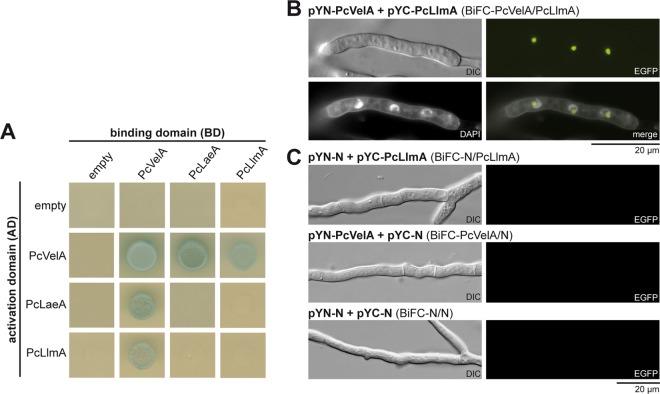
PcVelA directly interacts with the putative SAM-dependent MTase PcLlmA. (A) For yeast two-hybrid analysis, diploid yeast strains were spotted on selective media that lacked adenine and histidine (in order to select for *ADE2* and *HIS3*) and that were supplemented with X-α-Gal (to demonstrate *lacZ* reporter gene activity). (B) For BiFC analysis, genes encoding PcVelA and PcLlmA were fused to *eyfp* fragments encoding either the N or the C terminus of the yellow fluorescent protein, and strains harboring both constructs were analyzed using fluorescence microscopy. DAPI straining confirmed the nuclear localization of the PcVelA-PcLlmA interaction. (C) As a control, strains generating either both split EYFPs or one split EYFP together with EYFP-PcVelA/PcLlmA-EYFP are shown. Scale bar = 20 µm.

Next we focused on direct interactions between PcLlmA and components of the velvet complex. As shown in Fig. 3A, we confirmed that there was a direct interaction between PcVelA and PcLlmA using an *ex vivo* yeast two-hybrid (Y2H) approach. Briefly, diploid yeast strains that synthesize both the bait and the prey proteins were spotted on selective media that lacked adenine and histidine (in order to select for *ADE2* and *HIS3*) and that were supplemented with X-α-Gal (5-bromo-4-chloro-3-indolyl-α-d-galactopyranoside; to demonstrate *lacZ* reporter gene activity). Only those interactions that could be verified based on both reporter systems (growth on selective media and blue color) were considered positive. Interestingly, PcLlmA interacted with PcVelA but not with the other velvet components, i.e., PcVelB, PcVelC, PcVosA, or the putative MTase PcLaeA (Fig. 3A; Fig. S6). To confirm the interaction between PcVelA and PcLlmA *in vivo* and in the homologous system, we used bimolecular fluorescence complementation analysis (BiFC) (50). Genes encoding PcVelA and PcLlmA were fused to *eyfp* fragments encoding either the N or the C terminus of the yellow fluorescent protein (YFP), and strains harboring both constructs were analyzed using fluorescence microscopy. As a control, we investigated strains that generated only split enhanced YFPs (EYFPs) and strains producing one split EYFP together with either EYFP-PcVelA or PcLlmA-EYFP. As shown in Fig. 3B, strains carrying both Pc*velA-eyfp* and Pc*llmA*-*eyfp* fusion constructs showed clear EYFP signals, while no fluorescence was detectable in control strains (Fig. 3C). DAPI (4′,6-diamidino-2-phenylindole) staining demonstrated that the interaction between PcVelA and PcLlmA occurs in the nucleus (Fig. 3B), as was shown previously for the interaction between PcVelA and PcLaeA, PcVelB, PcVelC, and PcVosA as well as for the interaction of PcVelA with itself (6, 13).

10.1128/mSphere.00149-16.6 Figure S6  Interaction between PcLlmA and components of the velvet complex is restricted to PcVelA. For yeast two-hybrid analysis, diploid yeast strains were spotted on selective media lacking adenine and histidine and supplemented with X-α-Gal to demonstrate *ADE2* and *HIS3* as well as *lacZ* reporter gene activity. Download Figure S6, TIF file, 2.7 MB. Copyright © 2016 Becker et al. 2016 Becker et al. This content is distributed under the terms of the Creative Commons Attribution 4.0 International license.

### Functional characterization of the putative SAM-dependent MTase PcLlmA.

To further analyze the regulatory properties of PcLlmA, marker-free Pc*llmA* deletion, complementation, and overexpression strains were generated and verified using PCR, Southern blot, and qRT-PCR analyses. While the mutant strains showed no significant phenotypes in terms of penicillin biosynthesis efficiency or stress tolerance, there were marked differences in levels of conidiosporogenesis, pellet formation, and hyphal morphology compared to those of the wild-type strain. In all analyses, complementation of the deletion mutants with Pc*llmA* under the control of its native promoter sequence at its native locus restored the wild-type phenotypes.

**FIG 4:**
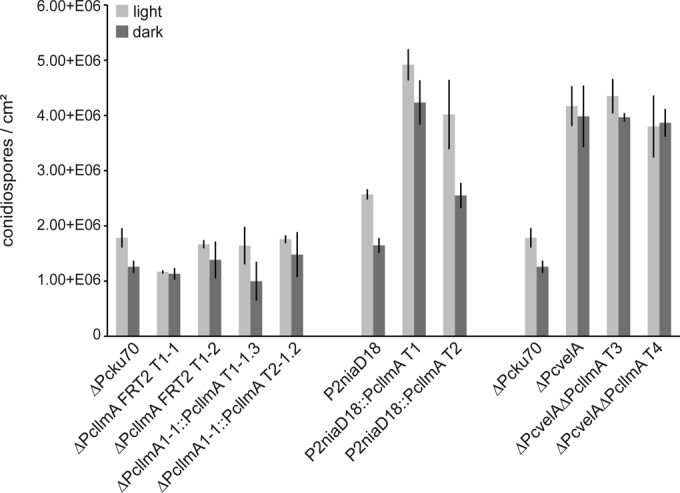
Quantitative analysis of conidiospore formation. Sporulation rates (numbers of conidiospores per cm^2^) are given for cultures grown for 120 h at 27°C under constant light (light bars) and constant dark (dark bars) conditions, respectively. Values are the mean scores from three biological replicates; averages ± standard deviations are indicated.

As shown in Fig. 4, ΔPc*llmA* strains showed almost no changes in their conidiospore formation rates compared to the corresponding wild-type ΔPc*ku70-*FRT2 strain under dark and light conditions. In contrast, the Pc*llmA* overexpression mutants were characterized by significantly elevated conidiospore formation compared to that of the corresponding wild-type strain P2niaD18, indicating that PcLlmA acts as a positive regulator of asexual development in *P. chrysogenum*. Conidiosporogenesis in these strains still showed light dependency, resulting in the formation of more spores when the organisms were grown under light versus dark conditions. These results are in line with previous studies of *A. nidulans*, demonstrating that overexpression of *llmF*, encoding a putative SAM-dependent MTase similar to PcLlmA, results in an increased formation of conidiospores but that deletion of *llmF* has no effect on asexual development in this fungus (40). To further test whether PcLlmA functions downstream of PcVelA, we investigated sporulation characteristics of ΔPc*velA* ΔPc*llmA* double mutants. The sporulation phenotype of these strains resembled the phenotype observed previously for the ΔPc*velA* strain, a strain that is characterized by elevated light-independent formation of conidiospores. These results support the idea that PcLlmA acts downstream of PcVelA.

**FIG 5:**
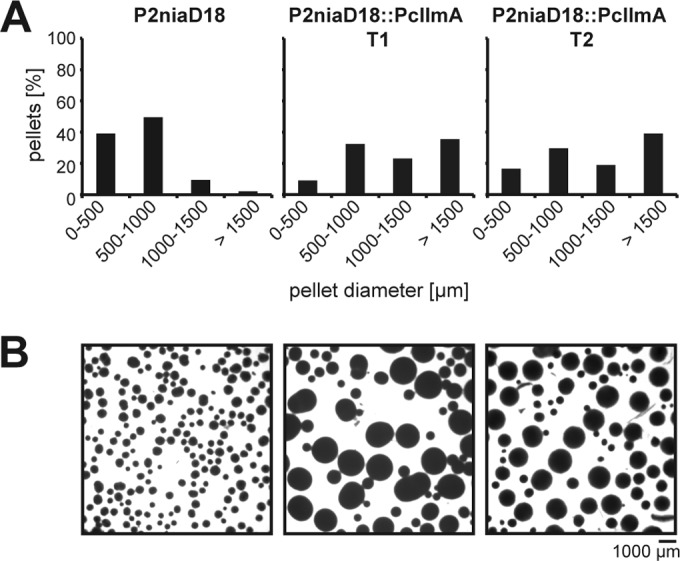
Quantification of pellet formation. (A) Distribution of pellet diameters after 72 h in liquid shaking cultures; (B) representative micrograph for each culture analyzed in panel A. Scale bar = 1,000 µm.

**FIG 6:**
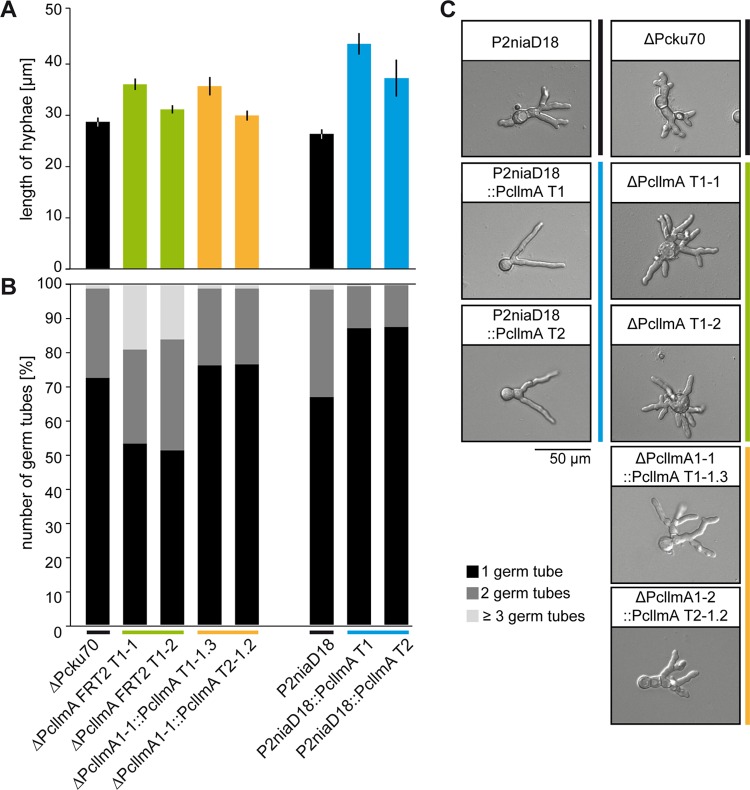
Hyphal morphology of germinating conidia. (A) Lengths of germinating hyphae were measured after 18 h of cultivation on solid CCM. Values are the mean scores of 300 independent measurements; averages ± standard deviations are indicated. (B) Numbers of germ tubes per germinating conidiospore were determined for 300 independent spores after 18 h of cultivation on solid CCM. Values are given as percentages of all analyzed hyphae per strain. Black, 1 germ tube; dark gray, 2 germ tubes; light gray, ≥3 germ tubes per conidiospore. (C) Representative micrographs of germinating conidiospores analyzed in panels A and B. Scale bar = 50 µm.

When pellet formation was analyzed in shaking cultures, again, we found that the deletion of Pc*llmA* did not result in specific phenotypic changes. However, Pc*llmA* overexpression led to a dramatic increase in pellet diameter (Fig. 5). About 35% of the pellets in these strains had diameters of ≥1,500 µm, whereas only ~2% of the pellets in the recipient P2niaD18 strain were that large. Notably, this phenotype resembled the one described previously for the ΔPc*velA* strain, which is characterized by the formation of larger and more-stable pellets than are formed by the corresponding wild-type strain (6). To better understand the origin of the observed phenotypic changes, we performed microscopic analysis of germinating conidiospores from Pc*llmA* overexpression and deletion strains as well as from the corresponding ΔPc*ku70-*FRT2 and P2niaD18 reference strains. As shown in Fig. 6A and C, Pc*llmA* overexpression mutants showed increased germ tube length, whereas ΔPc*llmA* strains showed no significant differences from the wild-type phenotype. However, when we focused on the number of hyphae that were emerging from the conidiospores, we found that the deletion strains were characterized by an increased formation of germ tubes with extensively branching tips. Specifically, ~20% of the germinating conidiospores formed ≥3 germ tubes versus ~2% in the recipient ΔPc*ku70-*FRT2 strain. In contrast, the overexpression mutants showed fewer germ tubes, and these tubes lacked terminal branching. Specifically, ~90% of the germinating conidiospores formed only 1 germ tube versus ~65% in the recipient P2niaD18 strain (Fig. 6B and C).

Taken together, these data show that PcLlmA controls not only asexual development but also pellet formation and the germination characteristics of *P. chrysogenum*. This observation is important, as a direct connection between putative MTases and hyphal and pellet morphology has been described for PcLaeA (6) but not for any other putative MTase in any other filamentous fungus until now.

## DISCUSSION

In recent years, characterization of the velvet complex components has been the focus of molecular genetic research in filamentous fungi (5 – 7). However, the molecular mechanisms underlying velvet-mediated regulation have remained unclear. In this report, we present the first ChIP-seq analysis of one of the core components of the velvet complex and provide evidence for the involvement of PcVelA in genome-wide transcriptional regulation at the DNA level. Furthermore, we introduce PcLlmA, a putative SAM-dependent MTase, as a downstream factor and direct interaction partner of PcVelA, which is involved in the regulation of developmental processes, such as conidiosporogenesis, pellet formation, and hyphal morphology.

### PcVelA acts as a regulator at the DNA level.

Most importantly, our work provides evidence that the regulatory functions of PcVelA are not restricted to protein-protein interactions with other velvet components but most likely include regulatory functions directly at the DNA level. In total, we identified 592 specific PcVelA-binding sites that showed distribution all over the *P. chrysogenum* genome. This observation is in agreement with an earlier hypothesis that the velvet proteins might act as global transcriptional regulators, representing a new fungus-specific class of TFs (16). Furthermore, our results are supported by recent data obtained with *H. capsulatum* and *A. nidulans* which demonstrated direct interaction between velvet proteins and DNA (18, 19, 51). ChIP-chip analysis of *H. capsulatum* revealed that Ryp2 and Ryp3, homologs of VosA and VeA/VelB, respectively, show genome-wide association with a total of 361 sites throughout the genome (18). Similarly, ChIP-chip and ChIP-PCR analysis proved that VosA from *A. nidulans* binds to more than 1,500 sites within the genome (19). ChIP-PCR analysis demonstrated further that VeA from *A. nidulans* binds DNA together with the blue-light TF LreA, in order to control light-dependent gene expression (51).

Using *in silico* prediction followed by EMSA, we identified a specific 10-nt PcVelA DNA-binding motif (PcVelA.M1), defined by the consensus sequence AACCTTGGAA, which was present in 46.5% of all PcVelA peak regions (*P* ≤ 0.001). The sequence shows strong similarity to the DNA-binding motif sequence described for the Ryp2/Ryp3 heterodimer (GAACCATGGT) (18) as well as moderate similarity to the DNA-binding motif sequence previously described for VosA (GCCTTGGCCAG) (19). Interestingly, comparison of PcVelA.M1 to the JASPAR CORE (2014) fungi database did not reveal any significant matches. Taken together, these observations provide further evidence for the idea that velvet proteins might represent a new class of fungal TFs which share similar DNA-binding properties. It remains unclear if or to what extent formation of homo- and heterodimers between velvet proteins and other factors affects their ability to specifically bind to DNA. For example, DNA-binding studies with Ryp2 and Ryp3 were successful only when a combination of both proteins was used, whereas both proteins alone did not show any binding to DNA. Similarly to that, *A. nidulans* VosA and VeA are able to bind DNA by themselves, whereas VelB binds as a heterodimer only with VosA *in vitro* (19). However, using ChIP-PCR analysis, Hedtke et al. (51) showed that VeA binding to DNA is dependent on phytochrome FphA, which, however, does not bind to DNA itself. VeA in turn is required for DNA binding of the blue-light TF LreA (51).

### The putative MTase PcLlmA acts as a downstream factor and interaction partner of PcVelA.

This study identified a remarkable number of putative PcVelA target genes that showed a direct association with processes that are regulated by velvet proteins, such as conidiation, development, and secondary metabolism. Interestingly, we also identified at least seven putative MTase genes as targets of PcVelA. A protein encoded by one of these genes, the putative SAM-dependent MTase PcLlmA, was identified as a downstream factor of PcVelA based on data obtained from ChIP-seq and qRT-PCR experiments. While overexpression of Pc*llmA* affected conidiosporogenesis, pellet formation, hyphal morphology, and germination characteristics, deletion of the gene resulted in phenotypic changes only within the context of conidiospore germination. Interestingly, the observed changes in asexual sporulation are not dependent on BrlA or PcVelA, as expression of the corresponding genes is not effected in the Pc*llmA* deletion and overexpression strains. As part of the functional characterization of PcLlmA, Y2H and BiFC analysis revealed direct interaction between PcVelA and PcLlmA at the protein level. This interaction seemed to be restricted to PcVelA, as no interactions between PcLlmA and other components of the velvet complex were detected. This observation is consistent with the results of previous Y2H analyses which found that the interaction between the putative MTase PcLaeA and components of the velvet complex was mediated solely by PcVelA (13). Furthermore, DAPI staining confirmed the nuclear localization of PcVelA-PcLlmA *in vivo*, which was described previously for PcVelA-PcLaeA and PcVelA-PcVelB (6), PcVelA-PcVelC and PcVelA-PcVosA (13), and VeA-LlmF (40), VeA-VipC, and VipC-VapB in *A. nidulans* (41). These observations suggest an important role for PcVelA in velvet-mediated regulatory functions in cooperation with a variety of putative MTases.

Notably, the interactions between VeA and putative MTases other than LaeA are not restricted to *P. chrysogenum*; rather, they seem to be a common feature of filamentous ascomycetes (52). For example, in *A. nidulans*, the LlmF MTase is involved in VeA localization, and the VipC and VapB MTases are involved in regulating sexual and asexual development (40, 41). In *Fusarium graminearum*, the velvet protein FgVeA interacts in a Y2H screen with a total of six putative MTases (42). It remains unclear how the interactions between VeA and the growing number of MTases are mediated at the structural level. Some suggest that VeA may have an affinity domain for MTases or a tertiary domain that interacts with MTases (5, 41). Experimental evidence is needed to confirm these hypotheses and to elucidate the functional consequences of these interactions in greater detail. Moreover, it will be highly interesting to see if PcLlmA has any protein MTase activity, which would make it one of the most promising PcVelA interaction partners identified so far. Similar functions have been hypothesized for LaeA and VapB, but experimental evidence for their direct involvement in protein methylation, let alone genome-wide chromatin modification, is lacking. When considering the functions of PcLaeA, VapB, and even PcLlmA at the molecular level, it should be noted that the biological roles of SAM-dependent MTases are versatile. Specifically, these proteins catalyze the transfer of methyl groups from SAM to a large variety of acceptor substrates that range from small metabolites to bio-macromolecules, including DNA, proteins, and secondary metabolites (42, 46, 53). On the one hand, this makes them highly interesting candidates for biotechnology applications (53); on the other hand, this emphasizes why researchers must consider the possibility that SAM-dependent MTases may have numerous functions besides those involved in the epigenetic modification of chromatin.

### Conclusions.

This study provides important insights into the regulatory functions of PcVelA on a genome-wide scale. The data presented here revealed that PcVelA is both a transcriptional regulator and a core component of the multisubunit velvet complex. The protein’s exceptional position as a scaffold that connects velvet proteins, putative MTases, and DNA will be investigated in greater detail in future studies. In addition, this work identified the putative MTase PcLlmA as a new interaction partner of PcVelA and as a regulator of conidiosporogenesis, pellet formation, and hyphal morphology in *P. chrysogenum*. Additional studies are needed to elucidate the molecular mechanisms underlying the regulatory functions that are mediated by this newly discovered MTase.

## MATERIALS AND METHODS

### Strains and culture conditions.

*Penicillium chrysogenum* strains (see Table S1 in the supplemental material) were grown in conditioned culture medium (CCM) (54) with shaking or as surface cultures at 27°C. For inoculation, we used 0.5 × 10^7^ spores derived from cultures grown on M322 solid medium (54) for 4 to 5 days. *Escherichia coli* strain XL1-Blue was used for cloning and plasmid propagation purposes, while BL21(DE3) served as a host for heterologous overexpression of PcVelA-GST (55, 56). *Saccharomyces cerevisiae* strains PJ69‑4a and PJ69-4α were used for yeast two-hybrid analysis (57). Strains were grown at 30°C on synthetic defined (SD) medium lacking selected amino acids used for auxotrophy marker selection. Mating of the PJ69‑4a and -α strains was performed in liquid yeast extract-peptone-dextrose agar (YPDA) medium at 30°C and 50 rpm.

10.1128/mSphere.00149-16.8 Table S1  *P. chrysogenum* strains used in this work. Download Table S1, PDF file, 0.05 MB. Copyright © 2016 Becker et al. 2016 Becker et al. This content is distributed under the terms of the Creative Commons Attribution 4.0 International license.

### Construction of *P. chrysogenum* strains.

Strains were constructed by ectopic or homologous integration of plasmid DNA (see Table S2 in the supplemental material) as described previously (6, 58), with some modifications. Recipient strains were grown for 72 h in shaking cultures, and protoplasts were transformed with either circular (for ectopic integration) or linear (for homologous recombination) plasmid DNA. Transformants were selected on CCM containing 150 µg/ml nourseothricin (Werner BioAgents, Germany). Resistant colonies were isolated and tested for integration of plasmid DNA. PCR analysis and SDS-PAGE–Western blot analysis were performed as described previously (6).

10.1128/mSphere.00149-16.9 Table S2  Plasmids used in this work. Download Table S2, PDF file, 0.03 MB. Copyright © 2016 Becker et al. 2016 Becker et al. This content is distributed under the terms of the Creative Commons Attribution 4.0 International license.

### Nucleic acid isolation, cDNA synthesis, qRT-PCR, and ChIP-PCR.

Isolation of nucleic acids, cDNA synthesis, qRT-PCR, and ChIP-PCR analysis were carried out as described earlier (25, 59, 60). A fragment of the 18S rRNA amplified using oligonucleotides SSU1 and SSU2 was used as a reference for normalization. Oligonucleotides are listed in Table S3.

10.1128/mSphere.00149-16.10 Table S3  Oligonucleotides used in this work. Download Table S3, PDF file, 0.02 MB. Copyright © 2016 Becker et al. 2016 Becker et al. This content is distributed under the terms of the Creative Commons Attribution 4.0 International license.

### Sample preparation for ChIP-seq, data analysis, and visualization.

ChIP and analysis of sequencing data were carried out as previously described (25), using Bowtie version 1.0.1 (61), SAMtools (62), the Integrative Genomics Viewer (IGV) (63), MEME (Multiple Expression Motifs [EM] for Motif Elicitation; http://meme-suite.org/) (64), TOMTOM (65), and the HOMER software for motif discovery and next-generation sequencing analysis (66).

### EMSAs.

Gel shift assays were performed using oligonucleotides derived from ChIP-enriched regions and purified GST-PcVelA_1–256_. Fifty-nucleotide double-stranded oligonucleotides (Table S3 were 5′-end labeled using polynucleotide kinase (Roche, Basel, Switzerland) and [γ-^32^P]ATP (Hartmann Analytic, Braunschweig, Germany). For shift experiments, 3.5- to 7.0-fmol (~50 to 100 cps) samples of radiolabeled oligonucleotides were incubated with various protein concentrations in the presence of 2 µl binding buffer (250 mM Tris-HCl, pH 8.0, 1 M KCl, 50% glycerol) and 1 µg poly(dI-dC)**-**poly(dI-dC) (Affymetrix USB, CA, USA) in a total volume of 20 µl for 20 min at room temperature. Samples were run on 5% polyacrylamide gels at 4°C in 190 mM glycine, 27 mM Tris-HCl, pH 8.5.

### Expression, purification, and immunodetection of recombinant PcVelA-GST protein.

Purification of recombinant PcVelA-GST protein from *E. coli* was performed as described earlier (67) using an elution buffer containing 50 mM Tris-HCl, 30 mM reduced glutathione, 100 mM NaCl, pH 8.0. Western blotting and immunodetection were performed using RPN1236 anti-GST horseradish peroxidase (HRP) conjugate (GE Healthcare, Germany).

### Yeast two-hybrid analysis.

Yeast two-hybrid analysis was carried out as described previously (13) using yeast strain PJ694a for GAL4 activation domain (AD) fusion derivatives and strain PJ69-4α for Gal4 DNA binding domain (BD) fusion constructs.

### Microscopy.

Fluorescence and light microscopy were carried out as described previously (6, 68) with minor modifications. Images were captured with a Photometrics CoolSNAP HQ camera (Roper Scientific, USA) and MetaMorph (version 7.7.5.0; Universal Imaging). Recorded images were processed with MetaMorph and Adobe Photoshop CS4/CS6. Staining of nuclei was performed using DAPI (Sigma Aldrich, Germany). For analysis of conidiospore germination, *P. chrysogenum* strains were grown on solid CCM for 5 days at 27°C. For each strain, three biological replicates were analyzed. For each replicate, 100 conidiospores were analyzed.

### Quantification of pellet diameter.

For analysis of pellet formation, *P. chrysogenum* strains were grown for 72 h at 27°C and 120 rpm in CCM shaking cultures. Pictures were taken at ×6.35 magnification, and interpretation was performed using the ImageJ software (http://imagej.nih.gov/ij/index.html).

### Quantification of conidiation.

Sporulation assays were performed as described previously (13) with some modifications. *P. chrysogenum* strains were grown on solid CCM, and incubation under light or dark conditions was performed for 120 h at 27°C.

### Accession number(s).

Raw sequencing data from ChIP-seq experiments are available from the NCBI SRA database under accession number SRP067220.
