# Supplementary figures and images for: Genome-Wide Chromatin Immunoprecipitation Sequencing Analysis of the Penicillium chrysogenum Velvet Protein PcVelA Identifies Methyltransferase PcLlmA as a Novel Downstream Regulator of Fungal Development
Source: mSphere. 2016 Jul 13;1(4):e00149-16. doi: 10.1128/mSphere.00149-16 (PMC4999599; doi:10.1128/mSphere.00149-16)

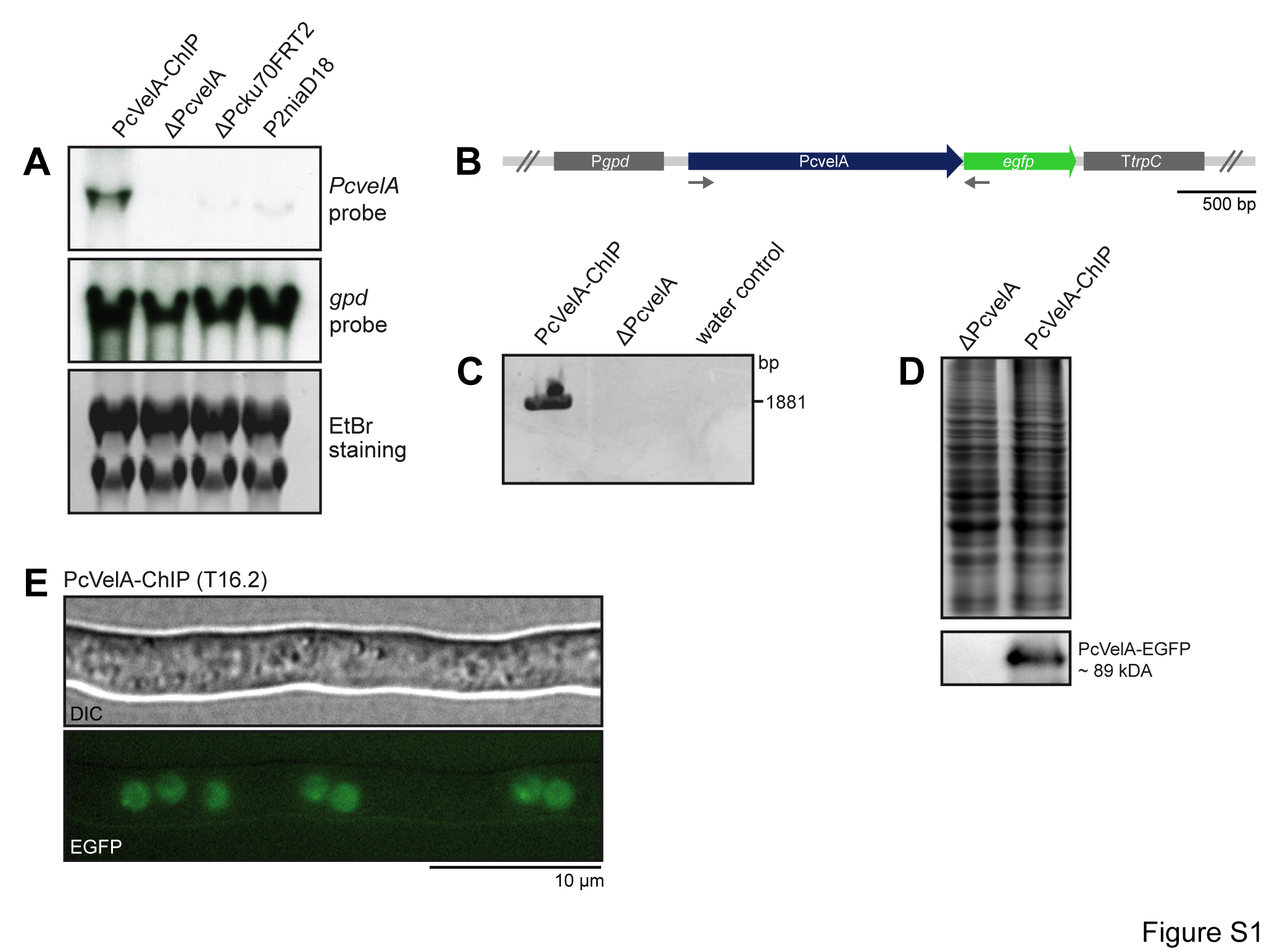

Supplement: Figure S1 [file sph004162112sf1.tif]

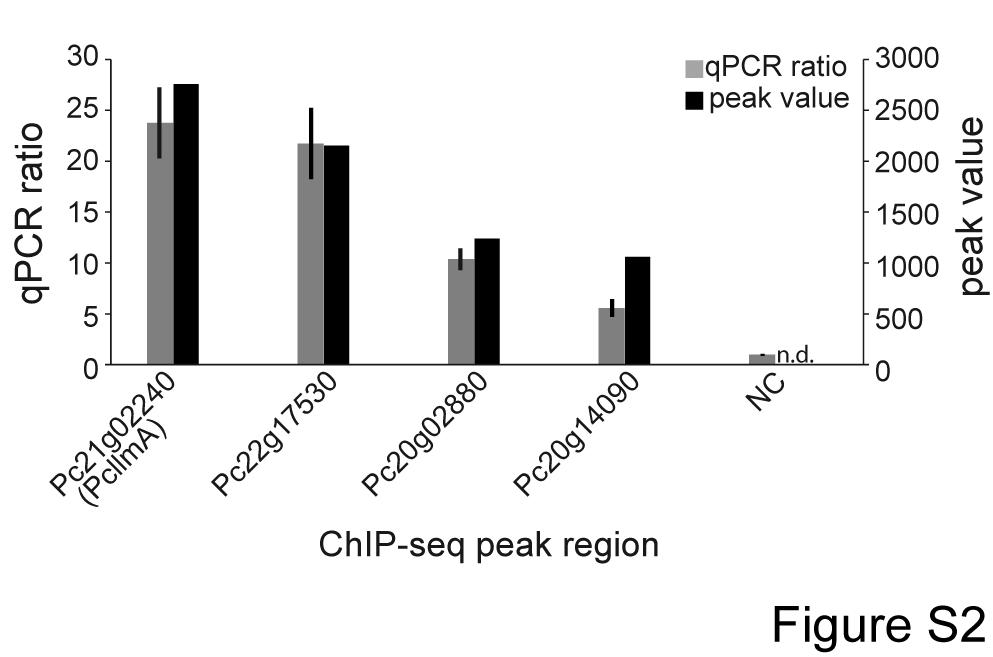

Supplement: Figure S2 [file sph004162112sf2.tif]

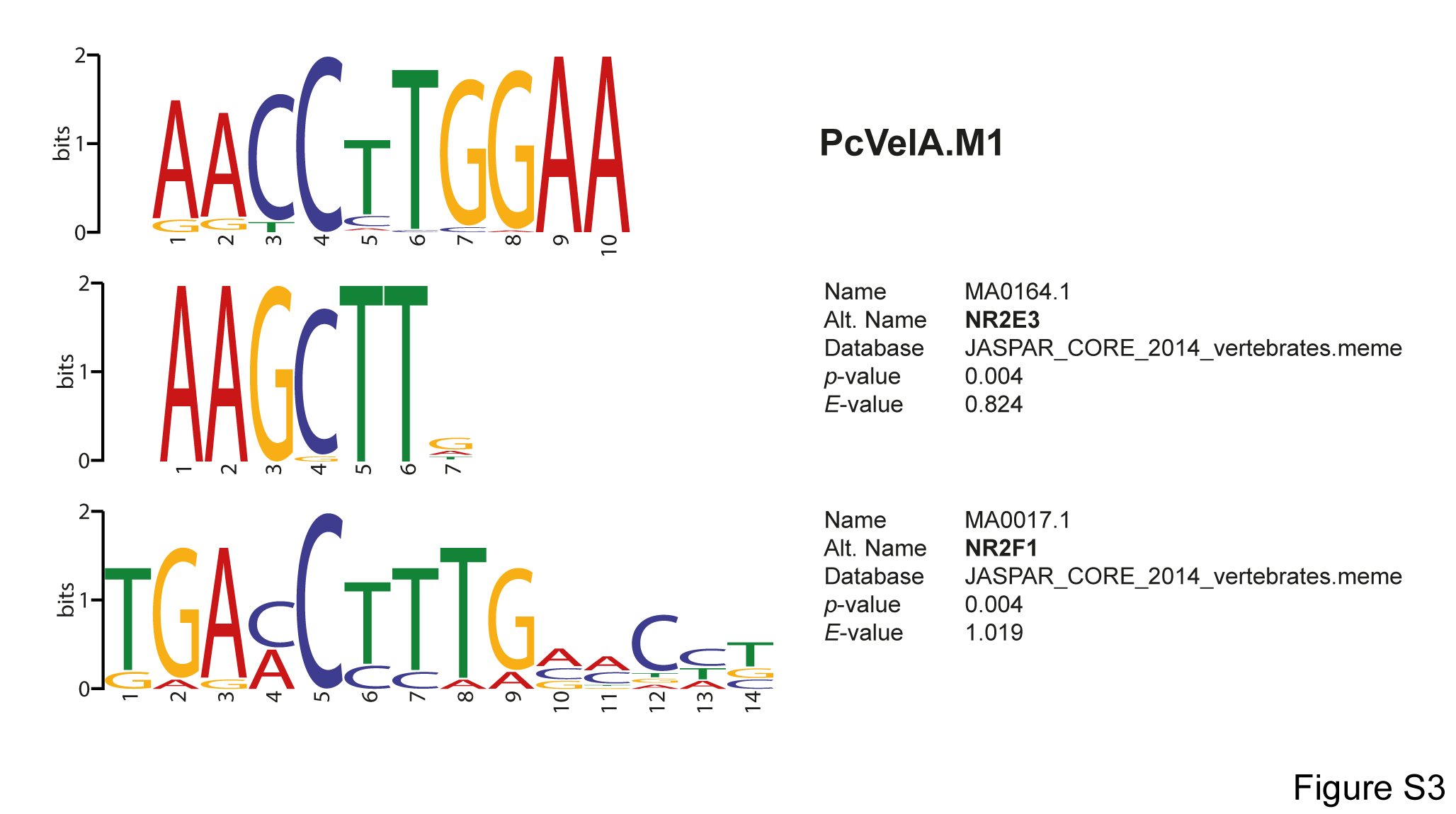

Supplement: Figure S3 [file sph004162112sf3.tif]

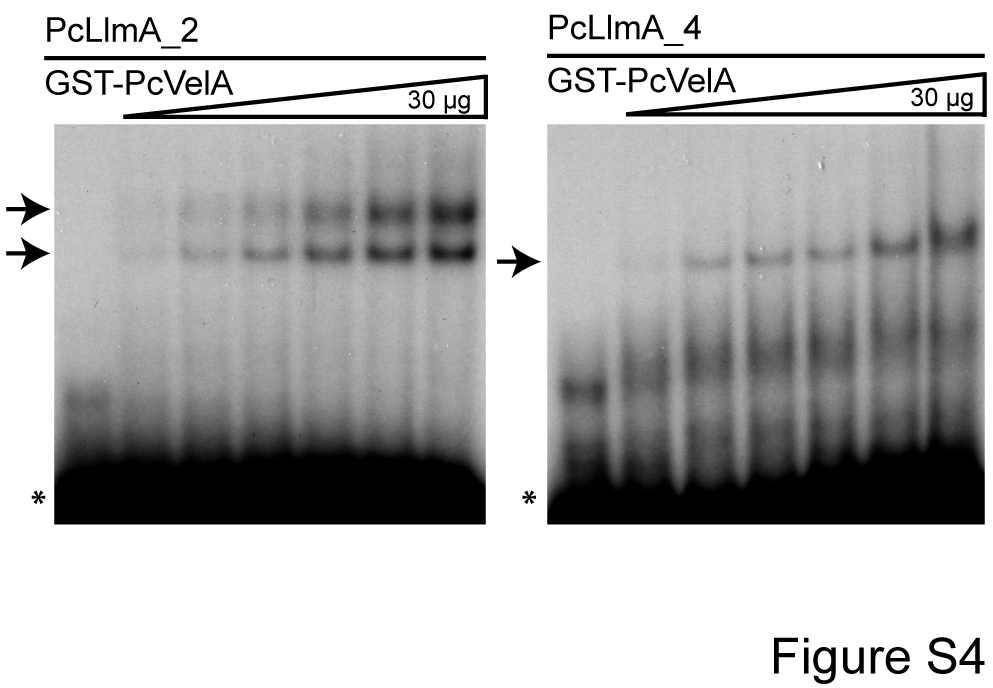

Supplement: Figure S4 [file sph004162112sf4.tif]

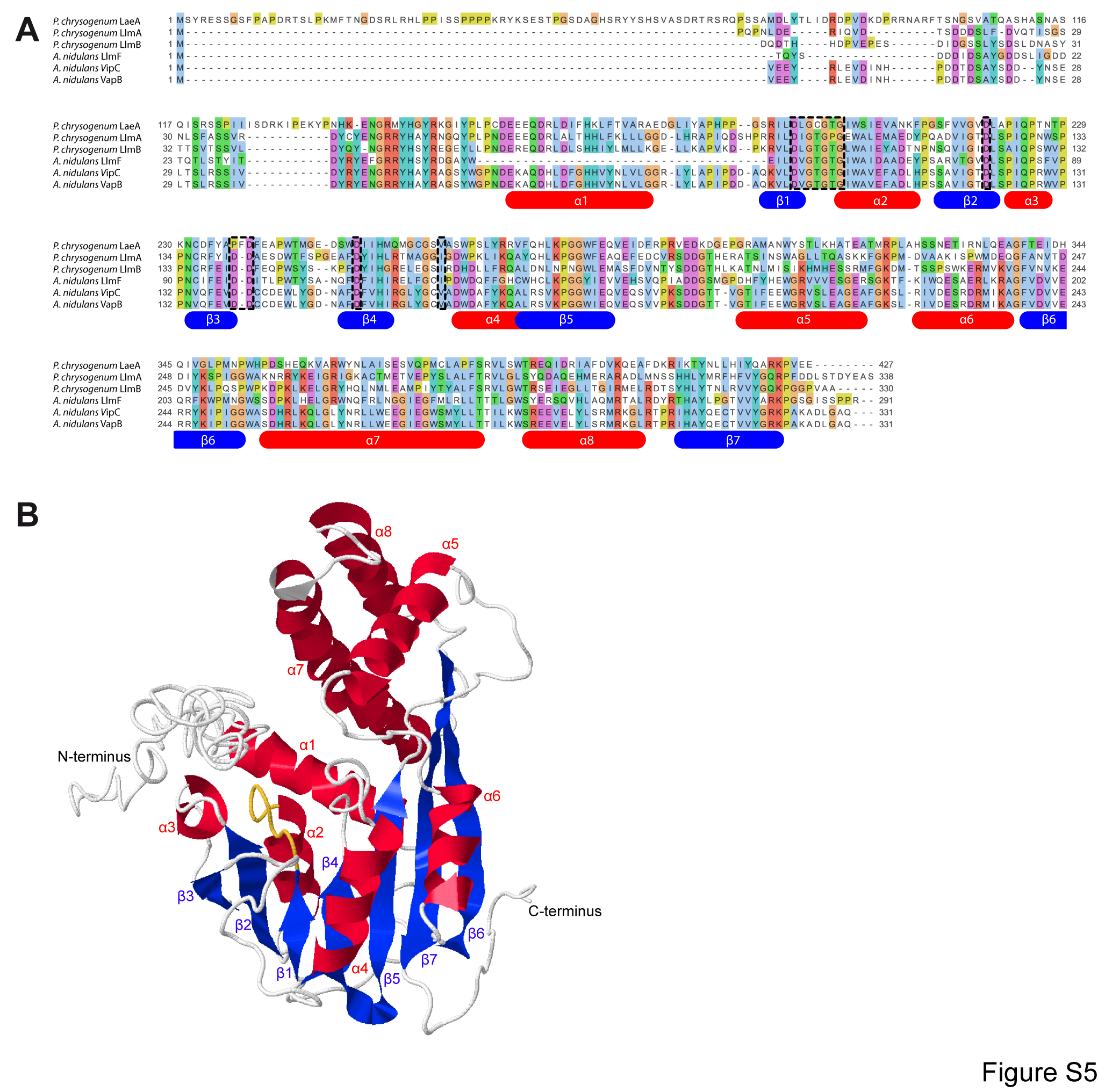

Supplement: Figure S5 [file sph004162112sf5.tif]

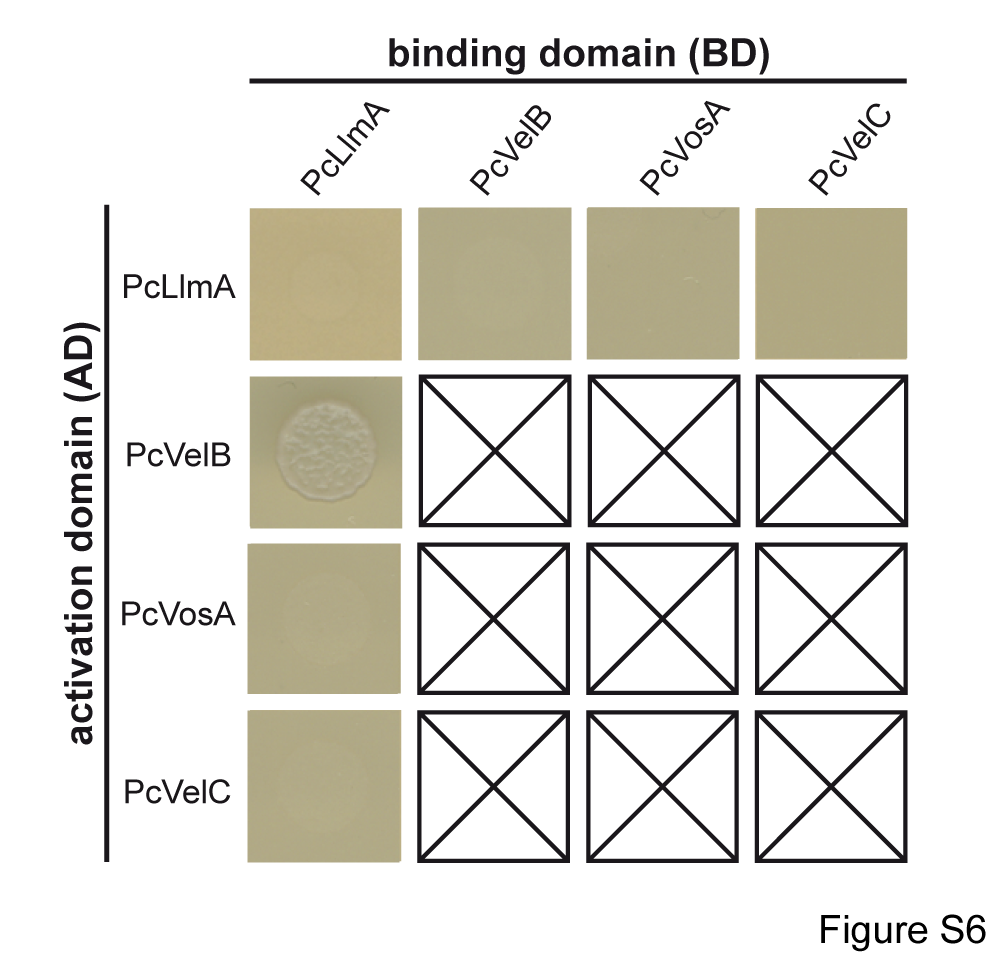

Supplement: Figure S6 [file sph004162112sf6.tif]
