# Supplementary material for: Genome-Wide Chromatin Immunoprecipitation Sequencing Analysis of the Penicillium chrysogenum Velvet Protein PcVelA Identifies Methyltransferase PcLlmA as a Novel Downstream Regulator of Fungal Development
Source: mSphere. 2016 Jul 13;1(4):e00149-16. doi: 10.1128/mSphere.00149-16 (PMC4999599; doi:10.1128/mSphere.00149-16)
Supplement: Table S1 [file sph004162112st8.pdf]

Table S1: *P. chrysogenum* strains used in this work

Strain	Characteristics and genotype	Source
P2niaD18	*niaD*⁻	(1)
ΔPcku70[b]	*Pcku70*Δ*::FRT; niaD*⁻	(2)
ΔPcvelA[b]	*Pcku70*Δ*::FRT; PcVelA*Δ*::FRT; niaD*⁻	(3)
PcVelA-ChIP (T16.2)	*Pcku70*Δ*::FRT; PcVelA*Δ*::FRT;* P_{gpd}*::PcVelA::EGFP::*T_{trpC}*;* P*trpC::nat1; niaD*⁻	This study
ΔPcllmAFRT1[a]	*Pcku70*Δ*::FRT; PcllmA*Δ*::FRT::*P*xyl::Pcflp::*P*trpC::nat1::FRT; niaD*⁻	This study
ΔPcllmAFRT2[b]	*Pcku70*Δ*::FRT*; *PcllmA*Δ*::FRT*; *niaD*⁻	This study
P2niaD18::PcllmA	P*gpd::PcllmA::*T*trpC*; P*trpC::nat1*; *niaD*⁻	This study
ΔPcllmAFRT2::PcllmA	*Pcku70*Δ*::FRT; PcllmA*Δ*::FRT*; P*PcllmA::PcllmA::*T*PcllmA;* P*trpC::nat1; niaD*⁻	This study
ΔPcvelAΔPcllmAFRT1	*Pcku70Δ::FRT; PcvelA*Δ*::FRT; PcllmA*Δ*::FRT::Pxyl::Pcflp::PtrpC::nat1::FRT; niaD*⁻	This study
BiFC-N/N	P*gpd::eyfpC::*T*trpC;* P*gpd::eyfp*N::T*trpC;* P*trpC::nat1; niaD*⁻	This study
BiFC-N/PcLlmA	P*gpd::eyfpC::*T*trpC; Pgpd::PcllmA::eyfpC::TtrpC*; *PtrpC::nat1; niaD*⁻	This study
BiFC-PcVelA/N	*Pgpd::PcvelA::eyfpN::TtrpC*; P*gpd::eyfp*N::T*trpC;* P*trpC::nat1; niaD*⁻	This study
BiFC-PcVelA/PcLlmA	*Pgpd::PcvelA::eyfpN::TtrpC*; *Pgpd::PcllmA::eyfpC::TtrpC*; *PtrpC::nat1; niaD*⁻	This study

[a] strains that still carry a resistance marker from the flipper knockout construct

[b] strains without any resistance markers due to FLP/*FRT* marker recycling.

1. **Hoff B, Pöggeler S, Kück U.** 2008. Eighty years after its discovery, Fleming's *Penicillium* strain discloses the secret of its sex. Eukaryot Cell **7:**465-470.
2. **Kopke K, Hoff B, Kück U.** 2010. Application of the *Saccharomyces cerevisiae* FLP/FRT recombination system in filamentous fungi for marker recycling and construction of knockout strains devoid of heterologous genes. Appl Environ Microbiol **76:**4664-4674.
3. **Kopke K, Hoff B, Bloemendal S, Katschorowski A, Kamerewerd J, Kück U.** 2013. Members of the *Penicillium chrysogenum* velvet complex play functionally opposing roles in the regulation of penicillin biosynthesis and conidiation. Eukaryot Cell **12:**299-310.
