# Supplementary material for: Genome-Wide Chromatin Immunoprecipitation Sequencing Analysis of the Penicillium chrysogenum Velvet Protein PcVelA Identifies Methyltransferase PcLlmA as a Novel Downstream Regulator of Fungal Development
Source: mSphere. 2016 Jul 13;1(4):e00149-16. doi: 10.1128/mSphere.00149-16 (PMC4999599; doi:10.1128/mSphere.00149-16)
Supplement: Table S2 [file sph004162112st9.pdf]

Table S2: Plasmids used in this work

Name	Characteristics	Source
pPcVelA-EGFP	P_{gpd} of *A. nidulans*, *egfp*, *PcvelA* gene of *P. chrysogenum*, T_{trpC} of *A. nidulans*, P*trpC* of *A. nidulans, nat* resistance gene of *Streptomyces noursei;* used for construction of *P. chrysogenum* ChIP-strains via ectopic integration into strain ΔPcVelA	This study
pEYFPC-nat	*gpd* promoter of *A. nidulans*, $eyfp_C$-fragment (aa 155-238), *trpC* terminator of *A. nidulans*, P*trpC* of *A. nidulans, nat* resistance gene of *Streptomyces noursei*	(1)
pEYFPN-nat	*gpd* promoter of *A. nidulans*, $eyfp_N$-fragment (aa 1-154), *trpC* terminator of *A. nidulan*s, P*trpC* of *A. nidulans, nat* resistance gene of *Streptomyces noursei*	(1)
pYNVELA	*PcvelA* ORF in *Not*I site of pEYFPN-nat	(1)
pYCLLMA	*PcllmA* ORF in *Nco*I and *Not*I site of pEYFPC-nat	(1)
pGADT7	*ADH1(p)::gal4 AD::LEU2*	Clontech
pGBKT7	*ADH1(p)::gal4 BD::TRP1*	Clontech
pAD-PcvelA	*PcvelA* cDNA in *Sma*I and *Sac*I site in pGADT7	(2)
pAD-PcllmA	*PcllmA* cDNA in *Eco*RI and *Bam*HI site in pGADT7	This study
pAD-PclaeA	*PclaeA* cDNA in *Eco*RI and *Xho*I site in pGADT7	(2)
pBD-PcvelA	*PcvelA* cDNA in *Sma*I and *Sac*I site in pGBKT7	(2)
pBD-PcllmA	*PcllmA* cDNA in *Eco*RI and *Bam*HI site in pGADT7	This study
pBD-PclaeA	*PclaeA* cDNA in *Eco*RI and *Pst*I site in pGBKT7	(2)
pKO_PcllmA	5' flanking region of *PcllmA* from *P. chrysogenum*, P*xyl* of *P. chrysogenum*, *flp* gene of *S. cerevisiae* (codon optimized for *P. chrysogenum*), P*trpC* of *A. nidulans*, *nat* resistance gene of *Streptomyces noursei*, *FRT* sites for marker recycling, 3' flanking region of *PcllmA* from *P. chrysogenum*	This study
pKL4	5' flanking region of *PcllmA* from *P. chrysogenum, PcllmA* of *P. chrysogenum,* P*trpC* of *A. nidulans*, *nat* resistance gene of *Streptomyces noursei,* 3' flanking region of *PcllmA* from *P. chrysogenum*	This study
pKL3	P_{gpd} of *A. nidulans, PcllmA* of *P. chrysogenum,* P*trpC* of *A. nidulans*, *nat* resistance gene of *Streptomyces noursei*	This study

1. **Hoff B, Kamerewerd J, Sigl C, Mitterbauer R, Zadra I, Kürnsteiner H, Kück U.** 2010. Two components of a velvet-like complex control hyphal morphogenesis, conidiophore development, and penicillin biosynthesis in *Penicillium chrysogenum*. Eukaryot Cell **9:**1236-1250.
2. **Kopke K, Hoff B, Bloemendal S, Katschorowski A, Kamerewerd J, Kück U.** 2013. Members of the *Penicillium chrysogenum* velvet complex play functionally opposing roles in the regulation of penicillin biosynthesis and conidiation. Eukaryot Cell **12:**299-310.
