# Supplementary material for: Genome-Wide Chromatin Immunoprecipitation Sequencing Analysis of the Penicillium chrysogenum Velvet Protein PcVelA Identifies Methyltransferase PcLlmA as a Novel Downstream Regulator of Fungal Development
Source: mSphere. 2016 Jul 13;1(4):e00149-16. doi: 10.1128/mSphere.00149-16 (PMC4999599; doi:10.1128/mSphere.00149-16)
Supplement: Table S3 [file sph004162112st10.pdf]

Table S3: Oligonucleotides used in this work

	Name	Sequence (5' to 3')	Specificity
Plasmid and strain construction	egfp_r	ACTTCAGGGTCAGCTTGC	*egfp* gene
	PcvelA_f	TCGGTCGACATGGCCAACAGACCATCTC	*PcvelA* gene
ChIP-PCR	qPCR_NC1_f	TTCTTCCGCAATCAAGCTCA	chr1:5375234-5375253
	qPCR_NC1_r	GAAAAATTGCCGCTGGACTC	chr1:5375364-5375383
	qPCR_NC2_f	GGTCGTTGATTCCCTTGAGC	chr2:7621179-7621198
	qPCR_NC2_r	GGATCGGATTATTCGGGTGA	chr2:7621294-7621313
	qPCR_Pc21g02240_f	CGAGAGAGAGGAACCCGGGA	chr2:5277047-5277066
	qPCR_Pc21g02240_r	TTTCCCGTACCAGGCTGTCG	chr2:5277176-5277195
	qPCR_Pc22g17530_f	AGGCACCGAAACCGTGAAGA	chr1:2788884-2788903
	qPCR_Pc22g17530_r	ACGCCAGGCCAGAGTTCAAT	chr1:2788799-2788818
	qPCR_Pc20g02880_f	CGTGAAATTCGAAGGTTCCCGA	chr2:2995008-2995029
	qPCR_Pc20g02880_r	AGAAATTAAGCCGCAAAACCCAGA	chr2:2994880-2994903
	qPCR_Pc20g14090_f	GTGGAAATTTCGGATGGGGTAGC	chr2:378180-378202
	qPCR_Pc20g14090_r	GATGCCCTGGTATCGGCAAAA	chr2:378053-378073
qRT-PCR	qRT_Pc21g02240_f	ACAAGGAAATCGGTCGCATC	chr2:5275851-5275870
	qRT_Pc21g02240_r	GCCCTCTCCATATGCTCCTG	chr2:5275747-5275766
	qRT_Pc18g01840_f	TTCGGCAAGGACATGACATC	chr1:5930185-5930204
	qRT_Pc18g01840_r	TGGTACCGACCAAGCTCCTT	chr1:5930361-5930380
	qRT_Pc21g12700_f	GGGTTTGTCGATACCCAGGA	chr2:7743588-7743607
	qRT_Pc21g12700_r	CCGCAGTCCACTGATGGTAA	chr2:7743677-7743696
	qRT_Pc18g04780_f	TGATGATGCGAAGACCATCC	chr1:6636202-6636221
	qRT_Pc18g04780_r	TGAACCAATGCACCAGCTCT	chr1:6636094-6636113
	qRT_Pc18g06010_f	CCCCGATACCAACGCATACT	chr1:6920825-6920844
	qRT_Pc18g06010_r	CGTGATCTTCAACCCAGCAG	chr1:6920718-6920737
	qRT_Pc13g15570_f	GGACCCGAACTCTGTTGCTC	chr4:2949445-2949464
	qRT_Pc13g15570_r	GCATCCACCACCTTCTCAAA	chr4:2949330-2949349
	qRT_Pc22g01170_f	TCGCTCGCTTCCTTGTATGA	chr3:5122377-5122396
	qRT_Pc22g01170_r	CAGGACTCGCAGACCAACAG	chr3:5122469-5122488
	SSU1	ATCCAAGGAAGGCAGCAGGC	SSUr RNA
	SSU2	TGGAGCTGGAATTACCGCG	SSUr RNA
EMSAs[a]	PcLlmA_2	TAGCGTCATTTATTTTTTTCTTCCAAGGTTTTTCCCTCTTCTTCGGAGT	chr2:5277074-5277122
	PcLlmA_2_m	TAGCGTCATTTATTTTTTTCcTtCAgaGTTTTTCCCTCTTCTTCGGAGT	chr2:5277074-5277122 (with mutations)
	PcLlmA_4	TCCGACAGCCTGGTACGGGAAACCTTGGAACCCATTCCAAATCGGTCTG	chr2:5277174-5277222
	PcLlmA_4_m	TCCGACAGCCTGGTACGGGAAACtcTGaAgCCCATTCCAAATCGGTCTG	chr2:5277174-5277222 (with mutations)

[a] in case of double-stranded oligonucleotides used for EMSAs, only the sense sequences are given.
